# A Mechanical Sensor Designed for Dynamic Joint Angle Measurement

**DOI:** 10.1155/2017/8465212

**Published:** 2017-03-14

**Authors:** Congo Tak-Shing Ching, Su-Yu Liao, Teng-Yun Cheng, Chih-Hsiu Cheng, Tai-Ping Sun, Yan-Dong Yao, Chin-Sung Hsiao, Kang-Ming Chang

**Affiliations:** ^1^Graduate Institute of Biomedical Engineering, National Chung Hsing University, Taichung, Taiwan; ^2^Department of Electrical Engineering, National Chi Nan University, Nantou, Taiwan; ^3^Department of Photonics and Communication Engineering, Asia University, Taichung, Taiwan; ^4^Department of Physical Therapy, Chang Gung University, Taoyuan, Taiwan; ^5^Department of Electronics Engineering, Nan Kai University of Technology, Nantou, Taiwan; ^6^Division of Science & Technology, Hong Kong Community College, Hong Kong, Hong Kong; ^7^Department of Medical Research, China Medical University Hospital, China Medical University, Taichung, Taiwan

## Abstract

*Background*. The measurement of the functional range of motion (FROM) of lower limb joints is an essential parameter for gait analysis especially in evaluating rehabilitation programs. *Aim*. To develop a simple, reliable, and affordable mechanical goniometer (MGR) for gait analysis, with six-degree freedom to dynamically assess lower limb joint angles. *Design*. Randomized control trials, in which a new MGR was developed for the measurements of FROM of lower limb joints. *Setting*. Reliability of the designed MGR was evaluated and validated by a motion analysis system (MAS). *Population*. Thirty healthy subjects participated in this study. *Methods*. Reliability and validity of the new MGR were tested by intraclass correlation coefficient (ICC), Bland-Altman plots, and linear correlation analysis. *Results*. The MGR has good inter- and intrarater reliability and validity with ICC ≥ 0.93 (for both). Moreover, measurements made by MGR and MAS were comparable and repeatable with each other, as confirmed by Bland-Altman plots. Furthermore, a very high degree of linear correlation (*R* ≥ 0.92 for all joint angle measurements) was found between the lower limb joint angles measured by MGR and MAS. *Conclusion*. A simple, reliable, and affordable MGR has been designed and developed to aid clinical assessment and treatment evaluation of gait disorders.

## 1. Introduction

Walking, as a characteristic of bipedalism, involves a multitude of body parts from the brain, the peripheral nervous system, and the musculatures to the feedback sensory system. Disturbance to the function of any parts would result in disorder of balance, speed, or pattern of walk. Clinically, gait analysis is an important tool used to determine the severity and cause of these disorders [1–5], with the aim of deciding and evaluating interventions [6].

Traditionally, observational gait analysis (OGA) is the simplest and most affordable approach to characterize gross abnormality by the naked eye. However, OGA can only provide qualitative, not quantitative, information on gait pattern and kinematics. The subjectivity of OGA lacks accuracy, good validity, reliability, sensitivity, and specificity [7] and relies on appropriate training and experience of the operators [8], especially as the complexity of walking pattern increases with age and pathological progressions. This calls for a more objective analysis, and instrumental gait analysis (IGA) provides a more accurate, sensitive, and reliable means of gait analysis to fulfill that role.

IGA is the current gold standard for objective evaluation of gait, through the use of advance technologies, such as video recordings and joint kinetic and kinematic measurements [9–12]. The objective and quantitative IGA, as a tool for gait pattern analysis, can enhance the effectiveness of clinically formulated management and intervention plans and their evaluations [13]. However, one drawback of the use of IGA is the high cost, as compared to OGA. An expensive gait laboratory with motion analysis system (e.g., Vicon) is a case in point. Apart from the substantial investment in expensive instruments [14], IGA also requires trained personnel, who on average need to spend about 3–6 hours on each assessment and the associate data interpretation [13]. On top of all the resource restriction, errors of up to 20 mm of upward displacement with respect to the underlying bones could be introduced by the skin-mount markers employed in the system in lower body kinematic measurements [15]. Though using flexible electrogoniometers could compensate for this drawback [16–19], they come with higher cost [20] and less durability. Similar to IGA with skin-mount marker, errors can be induced by electrogoniometers in estimating bone movements, influenced by the associated skin motion and tissue deformation [21–24].

To improve cost effectiveness and accuracy of clinical practices of gait kinematics analysis, ongoing investigation on available tools that are simple and cost effective is necessary. Amongst the various parameters of gait analysis, functional range of motion (FROM) is important for clinical gait assessment of lower limb joints in walking, especially for evaluation and comparison of the effectiveness of various treatment modalities [25, 26]. Clinically, only two-dimensional angular joint rotation on a single plane is considered in measurement of joint movements [27], and greatest deviations of progression were found in the sagittal plane movements of walkers with disorders [28]. Thus, the sagittal FROM is considered a more important parameter for clinical evaluations [28].

Mechanical goniometer (MGR) is a simple instrument that could be incorporated into IGA for measurements of functional range of motion, not only for objective and quantitative gait analysis, but also for simplicity and cost effectiveness. Though reported being employed in dynamic assessment of lower limb joint angles [29, 30], MGRs showed a limited degree of freedom. This study, therefore, aims to develop a simple, reliable, and affordable MGR with six-degree freedom for dynamic assessment of lower limb joint angles, to reduce cost and improve accuracy of gait analysis.

## 2. Materials and Methods

### 2.1. Architecture of the Designed MGR

**Figure 1:**
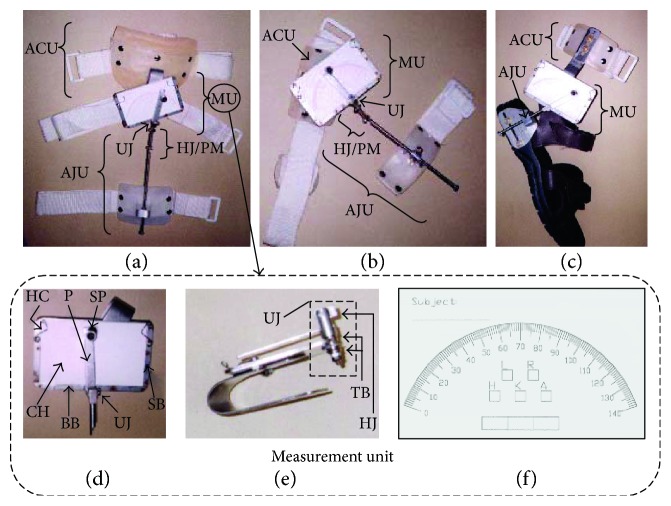
The MGR for dynamic assessment of lower limb joint angles of the (a) hip, (b) knee, and (c) ankle. The MGR is composed of 3 major parts: measurement unit, anchoring unit, and adjustment unit. The architecture of the measuring unit includes a measurement card, a cardholder, a universal joint, a pointer, and a pen. (d) The front view and (e) side view of the measuring unit. (f) The measurement card. Abbreviations: ACU stands for anchoring unit, MU for measurement unit, AJU for adjustment unit, UJ for universal joint, HJ/PM for the two hinge joints configured in parallelogram mechanism, P for pointer, SP for special pen, CH for cardholder, SB for side bars, BB for basement bar, HC for holding coils, HJ for hinge joint, and TB for thrust bearing permitting pivot rotation.

**Figure 2:**
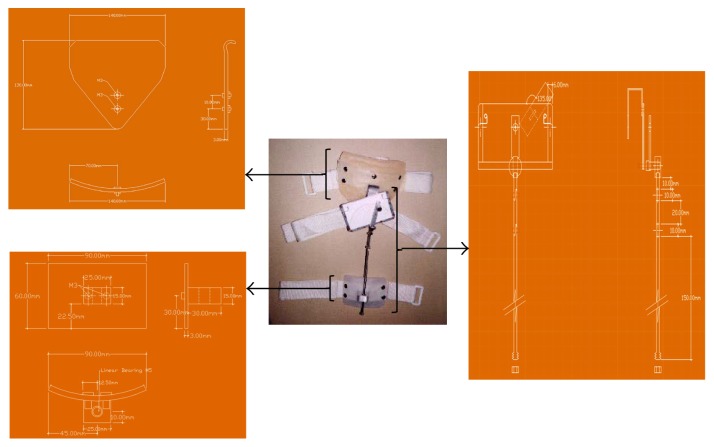
The mechanical drawing of the MGR for hip joint angle measurement.

**Figure 3:**
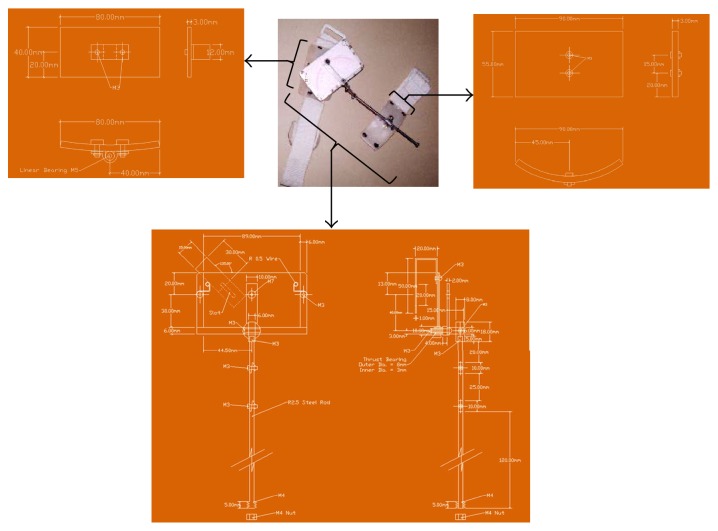
The mechanical drawing of the MGR for knee joint angle measurement.

**Figure 4:**
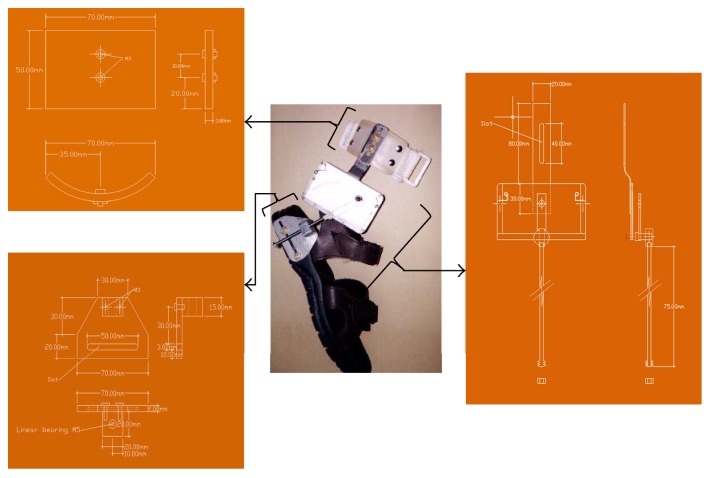
The mechanical drawing of the MGR for ankle joint angle measurement.

The designed MGR (Figures 1(a), 1(b), and 1(c)) is composed of 3 major parts: measurement unit, anchoring unit, and adjustment unit. The mechanical drawing of the MGR is shown in Figures 2–4.

#### 2.1.1. Measurement Unit

The measurement unit includes a measurement card, a cardholder, a universal joint, a pointer, and a pen (Figures 1(d), 1(e), and 1(f)). The measurement card (Figure 1(f)), with a scale of 0°–140° angulation in 1° increment, records FROM measurements of a joint. The size of the card is 90 mm × 60 mm. Spaces are provided in the card for the operator to fill in the subject's information, like subject name, the side of limb and joint to be tested, and the measurement result.

The cardholder (Figure 1(d)) houses the measurement card in operation and is composed of 2 side bars, a basement bar, and 2 movable holding coils.

The universal joint (Figure 1(e)) permits flexion and extension (permitted by thrust bearings allowing pivot rotation relative to the cardholder) of lower limbs between 0° and 180°, as well as abduction and adduction (permitted by hinge joint) between 0° and 90°. It is placed at the midbottom of the cardholder together with 2 thrust bearings to achieve frictionless motion (Figures 1(d) and 1(e)).

The pointer helps to position a custom-designed pen for recording of FROM of a joint on the measurement card. The pointer is attached to the universal joint (Figures 1(d) and 1(e)).

#### 2.1.2. Anchoring Unit

The anchoring unit, a curved surface to fit the body contour (Figures 1(a), 1(b), and 1(c)), provides points of attachment for the measurement unit, using a Velcro strap for suspension. The reason for using a Velcro strap is for the ease of donning and doffing.

#### 2.1.3. Adjustment Unit

Both adjustment units for the hip (Figure 1(a)) and knee (Figure 1(b)) joints consist of 4 components: a polypropylene shell, a metal block, a linear bearing, and a Velcro strap. During FROM measurement of the hip and knee joints, the adjustment unit is positioned at the thigh and shank regions, respectively. The polypropylene shell provides attachment for a metal block, holding the linear bearing. The linear bearing allows a metal rod, connected to the universal joint of the measurement unit through two hinges configured in parallelogram mechanism (Figures 1(a) and 1(b)), to pass through it permitting free sliding and rotation (0°–180°). The use of a universal joint, the hinge-joint parallelogram mechanism, and the linear bearing enables this designed MGR to possess six-degree freedom in flexion, extension, abduction, adduction, and rotation. The deployment of a Velcro strap keeps the adjustment unit fixed to the thigh and shank.

The adjustment unit for an ankle joint composes of 4 components: a metal plate, a metal block, a linear bearing, and a sandal (Figure 1(c)). The metal plate has a slot at its bottom, through which the metal plate attaches to the sandal (near the heel region) by screw, allowing it to slide back and forth. The function of the metal block and the linear bearing is the same as in the adjustment unit for hip and knee joints.

### 2.2. Subjects

Thirty healthy subjects (15 males and 15 females), aged 25 ± 6 years, participated in this study. Subjects had no history of lower limb musculoskeletal disorders and they had given written consent before the start of experiments. The study was approved by the Asia University Medical Research Ethics Committee.

### 2.3. Operators

Three operators participated in this study. Two of them had no previous experience in utilizing MGR. For these two operators, 10 minutes of basic training on the designed MGR has been provided before experiments.

### 2.4. Equipment

A motion analysis system (Vicon 370, Oxford Metrics, UK), MAS, with 6 CCD cameras was used for measuring the lower limb range of motion (ROM) in level walking. The lower limb ROM measurements obtained by using the MAS were taken as references to evaluate measurement of lower limb ROM generated by the designed MGR. The reason for using MAS to evaluate the designed MGR is that MAS is mainly and widely used in clinic. Moreover, MAS is principally the gold standard for lower limb joint angle measurement during gait analysis.

### 2.5. Experimental Procedures

Each participant was examined by 3 operators. For each operator, 3 sets of trial measurements were taken. And the designed MGR was removed from the leg and reapplied for each of these 3 trials. Before data collection, each participant was asked to walk with the MGR for 1 minute so that he/she can be familiar with the MGR during walking, in order to minimize the trial-to-trial variation.

**Figure 5:**
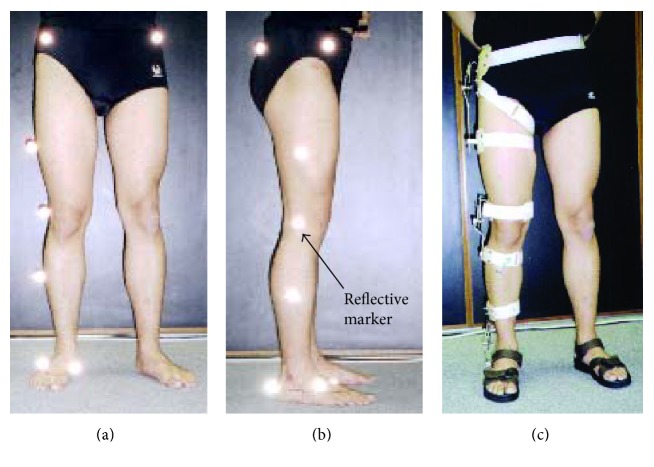
The position of the reflective markers (for MAS) fitted to a subject. (a) Front view and (b) side view of the reflective markers in positions. (c) The position of the MGR fitted to a subject for dynamic assessment of lower limb joint angles of the hip, knee, and ankle.

Fitted with reflective markers (for MAS) (Figures 5(a) and 5(b)) and the MGR (Figure 5(c)) to the lateral side of their right lower limbs, the subjects walked trice, along a 3-meter-long pathway, with normal speed and gait pattern. Data were recorded for each trial.

#### 2.5.1. Procedures of MGR Attachment

The procedure for the attachment of MGR for hip joint measurements (Figure 5(c)) is as follows: On the sagittal plane, the universal joint of the measurement unit was positioned 1/2 inch anterior and 1 inch superior to the apex of the greater trochanter. On the coronal and transverse planes, with the assistance of a simple bubble level meter, the cardholder of the measurement unit was positioned in parallel on a parasagittal plane. After positioning the MGR at a desired location, it was then fixated on the subjects by a Velcro strap.

The procedure for the attachment of MGR for knee joint measurements (Figure 5(c)) is as follows: On the sagittal plane, the universal joint of the measurement unit was positioned at the mid-distance between the medial tibial plateau and the adductor tubercle. Again, on the coronal and transverse planes, with the assistance of a simple bubble level meter, the cardholder of measurement unit was positioned on the parasagittal plane. After positioning the MGR at the desired position, it was fixated to the subjects by a Velcro strap.

The procedure for the attachment of MGR for ankle joint measurements (Figure 5(c)) is as follows: On the sagittal plane, the universal joint of the measurement unit was positioned at the apex of the lateral malleolus. Similarly, on the coronal and transverse planes, with the assistance of a simple bubble level meter, the cardholder of the measurement unit was positioned on the parasagittal plane. After positioning the MGR at the desired position, it was then fixated to the subjects by a Velcro strap.

#### 2.5.2. Data Collection from MGR

**Figure 6:**
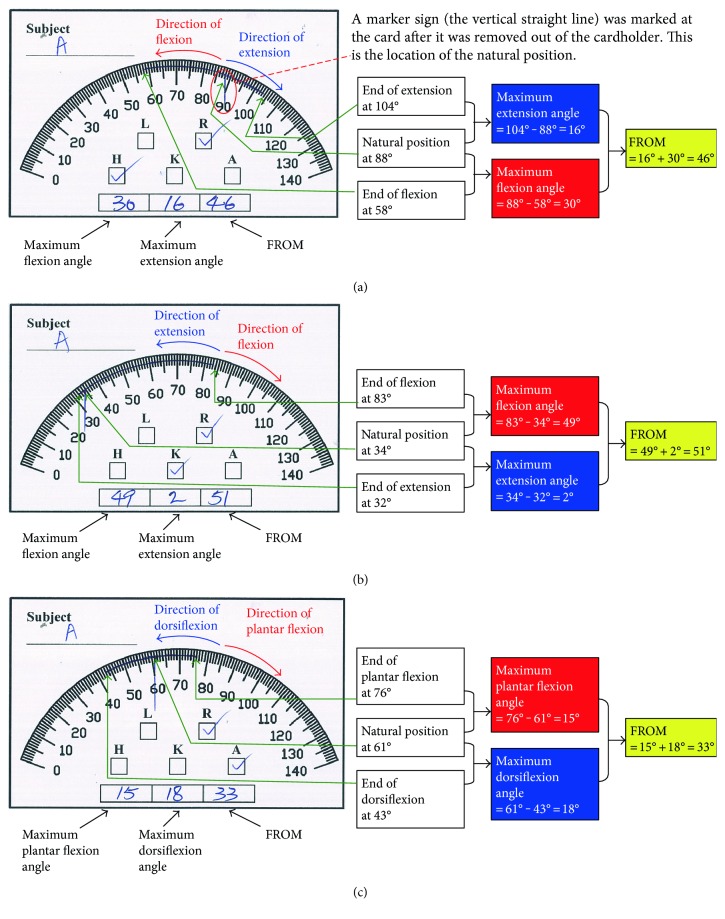
Data collection from the measurement card of MGR for dynamic assessment of lower limb joint angles: (a) right hip, (b) right knee, and (c) right ankle. The subject was asked to stand naturally and stationary after walking. Then the measurement card was removed from the cardholder and this removing process marked a sign onto the measurement card to indicate the location of the natural position. It should be borne in mind that the trajectory of flexion and extension of right lower limb joints are reversed in the left lower limb joints. For example, the right hip joint has a clockwise extension trajectory and anticlockwise flexion trajectory while the left hip joint has an anticlockwise extension trajectory and clockwise flexion trajectory. Abbreviations: L stands for left side, R for right side, H for hip, K for knee, and A for ankle.

Data collected on the measurement card of MGR is shown in Figure 6. On the measurement card, the maximum flexion, extension, and range of motion of joints can be recorded. In practice, subjects were asked to stand still naturally after walking, before removal of the measurement card from the cardholder, which marks a sign onto the measurement card to indicate the location of a natural position.

### 2.6. Statistical Analysis

Intraclass correlation coefficient (ICC) was used to evaluate inter- and intrarater reliability for the measurement of the hip, knee, and ankle joint angles measured by the designed MGR. ICC (3,k) was determined to test the intrarater reliability, made by comparing the 3 sets of trial measurements taken by an operator. Concurrently, ICC (2,k) was determined to test the interrater reliability, made by comparing measurements taken by the 3 individual operators. In addition, measurements obtained by the MGR and MAS were used to draw a Bland-Altman plot to compare the variability between measurements taken by the two. Moreover, linear regression analysis was used to evaluate relationship between measurements of lower limb joint angles generated by MGR and MAS. All statistical analyses were carried out using SPSS v.11.5 software (SPSS Inc., Chicago, Illinois, USA) with the level of significance set at 0.05.

## 3. Results

**Figure 7:**
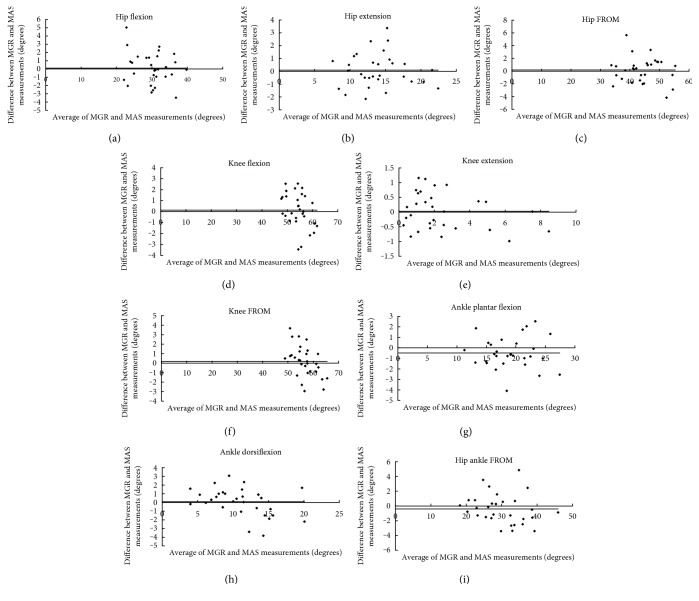
Bland-Altman plot of the joint motion measured by MGR and MAS: (a) hip flexion, (b) hip extension, (c) hip FROM, (d) knee flexion, (e) knee extension, (f) knee FROM, (g) ankle plantar flexion, (h) ankle dorsiflexion, and (i) ankle FROM. Almost all data points were within range of ±2 standard deviation lines, and hence measurements made by MGR and MAS were agreeable and repeatable. Remarks: dots represent the “difference”; solid lines represent the “mean difference”; dotted lines represent the “mean difference ± 2 standard deviation lines.”

ICC for the inter- and intrarater reliability for the measurement of the hip, knee, and ankle joint angles taken by MGR are summarized in Table 1. All joint-angle-motion measurements, for both inter- and intrarater, had ICC value ranging from 0.93 to 0.99 with *p* < 0.001 for all. On the other hand, Bland-Altman plots (see Figure 7) showed that almost all measurement points were located within the ±2 standard deviation lines.

**Figure 8:**
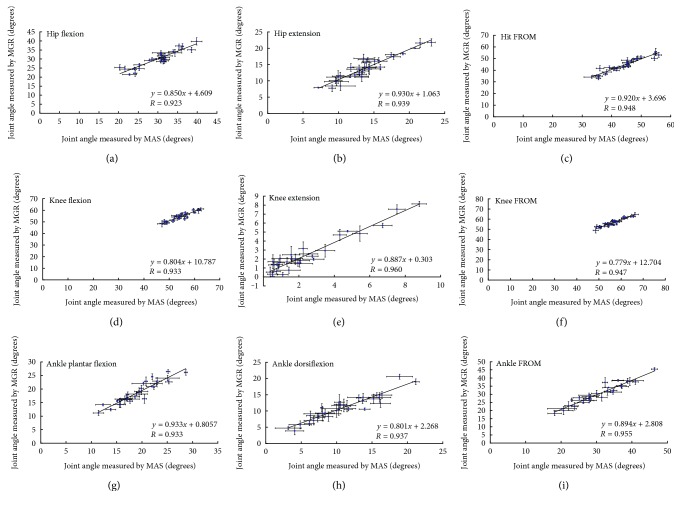
The linear correlations between joint motion measured by MGR and MAS: (a) hip flexion angles, (b) hip extension angles, (c) hip FROM, (d) knee flexion angles, (e) knee extension angles, (f) knee FROM, (g) ankle plantar flexion angles, (h) ankle dorsiflexion angles, and (i) ankle FROM. The line is of best fit found by linear regression. Establishing the statistical significance (*p* < 0.001 for all measurements) of the linear regression (*R* ≥ 0.92 for all measurements), the model could be used to predict outcome variables with good statistical significance. Results (*n* = 30) are expressed as mean and standard deviation.

The linear correlations of the joint angles measured by MGR and MAS were quantified by the correlation coefficient *R*. A very high degree of linear correlation (*R* ≥ 0.92 and *p* < 0.001 for all joint angle measurements) was found to exist between the lower limb joint angles measured by MGR and MAS (Figure 8 and Table 2). In all cases, at least 85% of measurements from MGR and MAS (*R*^2^ ≥ 0.85) could be explained (Table 2).

## 4. Discussion

In this study, a new MGR was designed and developed, which is simple and affordable. The MGR provides direct readout of the maximum joint angles recordable during walking. The operation of the MGR is simple in that only a quick 10 min training is needed for an operator to master the attachment of MGR to subjects as described in Section 2.5.1 and data collection from the measurement card of MGR as described in Section 2.5.2. On average, the operators only spent approximately 8 minutes to measure 4 trials of the maximum flexion, extension, and range of motion of joints, including donning and doffing of the MGR. Therefore the new MGR is also less time consuming and more effective to operate.

When using this new MGR, possible out-of-plane motion (i.e., abduction, adduction, and rotation) of the joints measured could be accommodated by the universal joint on the measurement unit of the MGR. Additional out-of-plane motion would also be accommodated by a parallelogram mechanism in the measurement (Figures 1(a), 1(b), and 1(c)). The MGR, is thus demonstrated to be capable of allowing six-degree freedom of movements, including flexion, extension, abduction, adduction, and rotation, in addition to its simple operation and cost effectiveness.

Reduction on maintenance cost of the MGR can be achieved through the use of a thrust bearing (Figure 1(e)) in hinge joint of the measurement unit, substantially reducing wear and tear and increasing lifespan of the joint. By using Velcro strap for fixation of the MGR on patients' lower limbs, the donning and doffing procedures could be simplified, which greatly shortens operation time (8 minutes for 4 trial measurements including donning and doffing of the MGR).

Moreover, the cost of our designed MGR is about US$66, a very low figure compared to a flexible electrogoniometer [31] and MAS [32]. Although the use of both flexible electrogoniometers [16–19] and MAS [33–36] is common for IGA, they are costly [14, 20, 31, 37, 38]. For example, a flexible electrogoniometer would cost about US$700 [31] for the sensor alone, with additional cost for accompanying cables and a transducer amplifier. Similarly, a MAS (Vicon MX) would come with high cost at about US$420,000 [32]. Therefore, the affordable price of this newly designed MGR would greatly facilitate its clinical applications, especially in developing countries like those in Africa. More importantly, a flexible electrogoniometer could easily be damaged through improper use (i.e., nondurable) while the maintenance fee for a MAS is expensive, not to mention operation of MAS definitely requires trained and skillful operators (with average 30 hrs training) before accurate analysis of results made possible. Therefore, this newly designed MGR may be a cost effective alternative for clinical use in both the developed and developing countries.

Though only providing information on maximum flexion, extension, and range of motion of joints, the new MGR is adequate in obtaining information for clinicians, for example, physician, physiotherapists, and prosthetist-orthotists, to evaluate cases and make clinical decisions with ease and cost effectiveness.

Reliability and validity of the new MGR are confirmed through ICC, which quantifies the reproducibility of a variable and also measures the homogeneity within groups of repetitive measurements relating to the total variation between groups. Statistically, ICC values ranging from 1.0 to 0.81 are considered excellent in reliability; from 0.80 to 0.61 very good; from 0.60 to 0.41 good; from 0.40 to 0.21 reasonable; and from 0.20 to 0.00 poor [39]. Reliability should also exceed 0.90 to ensure reasonable validity for many clinical measurements [39]. In this study, all the ICC measurements for both inter- and intrarater exceeded 0.90 (Table 1), implying both good reliability and reasonable validity. Thus, this newly designed MGR is fit for clinical use in measuring joint angles with good reliability and reasonable validity in general, within patients and between patients.

The accuracy of the new MGR is also tested through the use of the Bland-Altman plots, showing almost all data points located within a range of ±2 standard deviation lines (Figure 7). Specifically, no point beyond the ±2 standard deviation lines was observable in knee extension (Figure 7(e)). One out of 30 (3.3%) points was located beyond the ±2 standard deviation lines as in hip flexion (Figure 7(a)), hip extension (Figure 7(b)), knee FROM (Figure 7(f)), and ankle FROM (Figure 7(i)). Two out of 30 (6.7%) points were beyond the ±2 standard deviation lines in hip FROM (Figure 7(c)), knee flexion (Figure 7(d)), ankle plantar flexion (Figure 7(g)), and ankle dorsiflexion (Figure 7(h)). Based on the Bland-Altman plots (Figure 7), measurements made by MGR and MAS were agreeable and repeatable. Therefore, this suggested that the low-cost MGR can substitute the expensive MAS, the clinical gold standard instrument, for lower limb joint angle measurement in the sagittal plane during clinical gait analysis.

Further confirmation of the reliability and validity of the MGR was shown in the linear correlation study (Table 2), where a very high degree of linear correlation (*R* ≥ 0.92 for all joint angle measurements) with statistical significance (*p* < 0.001 for all joint angle measurements) were found between the lower limb joint angles measured by MGR and MAS (Figure 8). As shown in the *R*^2^ (i.e., coefficient of determination) column of Table 2, at least 85% of the lower limb joint angles, measured by MGR and MAS, could be explained. Based on the established the statistical significance of the regression model, the outcome variable with statistical significance could be predicted. MAS contributes significantly (*p* < 0.001 for all) to the regression model for all joint angle measurements but the (constant) only contributes significantly to the regression model for several joint angle measurements (see *p* value column under Coefficients in Table 2). By analyzing the B column under Unstandardized Coefficients in Table 2, the regression equations could be established as shown in Figure 8. Taking in all consideration previously stated, it could therefore be concluded that the designed MGR is accurate in measuring joint angles of lower limbs in walking. Hence, this further suggested that the MGR can substitute the MAS for lower limb joint angle measurement in the sagittal plane during clinical gait analysis. Since the MGR is accurate, affordable, and durable, it would greatly facilitate its clinical applications, especially in developing countries like Africa.

The potential clinical applications of the designed MGR could range from evaluation of gait pathology to design of therapeutic interventions. For example, physiotherapists could use it to investigate the range of motion of a patient before and after tendon stretching (e.g., tendo calcaneus) and/or to monitor treatment progression. Clinicians could use it to simply and economically quantify the functional status of a patient who has undergone hip replacement as part of routine clinic practice. Similarly, prosthetist-orthotists and physiotherapists could use it to train an amputee to achieve a symmetric gait pattern with the use of prosthesis. Moreover, prosthetist-orthotists could use it to evaluate the effectiveness of a knee brace controlling knee hyperextension, as well as to investigate the gait pattern of a stroke patient fitted with an ankle-foot orthosis to control foot drop.

It is our goal to further evaluate the designed MGR for its applications on patients' gait analysis after stroke, cerebral palsy, or any other more serious motor disorders.

## 5. Conclusion

A new mechanical goniometer with six-degree freedom, for dynamic assessment of lower limb joint angles, has been designed and developed and reported here. The new MGR is simple, reliable, user friendly, and affordable, with many potential clinical applications. It could provide direct readout of the maximum joint angles and functional range of joint motion in walking without computation. The simple operation enables the operator, with only a 10-minute training, to complete 4 measurements in 8 minutes. Moreover, material cost for one set of the designed MGR (included hip, knee, and ankle unit for bilateral side) is about US$66, a very low figure compared to current commercially available tools with comparable functionalities.

## Figures and Tables

**Table 1:** Intraclass correlation coefficients (ICC) for studying the intra- and interrater reliability of raters in measuring the hip, knee, and ankle joint angles using the designed MGR.

Measurement of joint angle motion		ICC			
		Intrarater reliability^a^		Interrater reliability^b^	
		ICC (3,k)	*p* value	ICC (2,k)	*p* value
Hip	Flexion	0.97	<0.001	0.99	<0.001
	Extension	0.94	<0.001	0.94	<0.001
	FROM	0.95	<0.001	0.95	<0.001

Knee	Flexion	0.94	<0.001	0.95	<0.001
	Extension	0.95	<0.001	0.93	<0.001
	FROM	0.99	<0.001	0.97	<0.001

Ankle	Plantar flexion	0.97	<0.001	0.95	<0.001
	Dorsiflexion	0.95	<0.001	0.96	<0.001
	FROM	0.97	<0.001	0.94	<0.001

^a^One rater involved in the evaluation of intrarater reliability.

^b^Three raters involved in the evaluation of interrater reliability.

**Table 2:** Linear regression analysis between lower limb joint angles measured by MGR and MAS.

Joint angle measurement	Pearson correlation coefficient, *R*	Coefficient of determination, *R*^2^	*p* value	Coefficients			
				Linear regression model	Unstandardized coefficients		*p* value
					*B*	Std. error	
Hip flexion	0.923	0.851	<0.001	(Constant)	4.609	2.042	0.032
				MAS	0.850	0.067	<0.001
Hip extension	0.939	0.882	<0.001	(Constant)	1.063	0.927	0.261
				MAS	0.930	0.064	<0.001
Hip FROM	0.948	0.899	<0.001	(Constant)	3.696	2.592	0.165
				MAS	0.920	0.058	<0.001

Knee flexion	0.933	0.870	<0.001	(Constant)	10.787	3.190	0.002
				MAS	0.803	0.059	<0.001
Knee extension	0.960	0.921	<0.001	(Constant)	0.303	0.160	0.068
				MAS	0.887	0.049	<0.001
Knee FROM	0.947	0.896	<0.001	(Constant)	12.704	2.845	<0.001
				MAS	0.779	0.050	<0.001

Ankle plantar flexion	0.933	0.870	<0.001	(Constant)	0.806	1.342	0.553
				MAS	0.933	0.068	<0.001
Ankle dorsiflexion	0.937	0.878	<0.001	(Constant)	2.268	0.665	0.002
				MAS	0.801	0.056	<0.001
Ankle FROM	0.955	0.913	<0.001	(Constant)	2.808	1.619	0.094
				MAS	0.894	0.052	<0.001
